# High-Sensitivity Microwave Sensor Based on an Interdigital-Capacitor-Shaped Defected Ground Structure for Permittivity Characterization

**DOI:** 10.3390/s19030498

**Published:** 2019-01-25

**Authors:** Junho Yeo, Jong-Ig Lee

**Affiliations:** 1 School of Computer and Communication Engineering, Daegu University, 201 Daegudae-ro, Gyeongsan, Gyeongbuk 38453, Korea; 2 Department of Electronics Engineering, Dongseo University, San69-1, Jurye-2dong, Sasang-gu, Busan 47011, Korea; leeji@dongseo.ac.kr

**Keywords:** high-sensitivity, microwave sensor, interdigital-capacitor-shaped, defected ground structure, permittivity characterization

## Abstract

This study proposes a high-sensitivity microwave sensor based on an interdigital-capacitor-shaped defected ground structure (IDCS-DGS) in a microstrip transmission line for the dielectric characterization of planar materials. The proposed IDCS-DGS was designed by modifying the straight ridge structure of an H-shaped aperture. The proposed sensor was compared with conventional sensors based on a double-ring complementary split ring resonator (CSRR), a single-ring CSRR, and a rotated single-ring CSRR. All the sensors were designed and fabricated on 0.76-mm-thick RF-35 substrate and operated at 1.5 GHz under unloaded conditions. Five different standard dielectric samples with dielectric constants ranging from 2.17 to 10.2 were tested for the sensitivity comparison. The sensitivity of the proposed sensor was measured by the shift in the resonant frequency of the transmission coefficient, and compared with conventional sensors. The experiment results show that the sensitivity of the proposed sensor was two times higher for a low permittivity of 2.17 and it was 1.42 times higher for a high permittivity of 10.2 when compared with the double-ring CSRR-based sensor.

## 1. Introduction

The response of a material to electromagnetic (EM) waves depends on its permittivity. Therefore, the accurate measurement of the permittivity is crucial for the design of antennas, microwave circuits, and non-destructive monitoring applications [1,2]. In general, permittivity measurement methods can be classified into resonant and non-resonant methods. In non-resonant methods, the permittivity property of the material is derived from changes in the characteristic impedance and wave velocity of EM waves, measured by the reflection and transmission characteristics. These methods require a means of directing the EM wave towards a material under test (MUT) and then collecting what is reflected and transmitted through it. The non-resonant methods include the waveguide and coaxial transmission line method [3,4,5], free space transmission method [6,7], open-ended transmission line method [8,9], and planar transmission line method [10,11].

The resonant methods can be divided into resonator and resonant perturbation methods [1]. In resonant perturbation methods, the MUT is inserted into a resonant structure, which causes a perturbation in the response in the form of a resonant frequency shift and a quality factor change [12,13,14]. However, these methods require a bulky and costly cavity such as a waveguide cavity or a coaxial cavity for the measurement. In resonator methods, the MUT is considered as a part of the resonator, and the permittivity can be deduced from the shift in resonance frequency [2,15,16].

For resonator methods, planar resonators based on split ring resonator (SRR) [16,17,18,19,20] and complementary SRR (CSRR) [2,21,22,23,24,25,26,27] structures have been widely used because of their advantages such as simple geometry, ease of fabrication, and low cost. For CSRR-based permittivity sensors, conventional CSRR structures using double rings or a single ring were etched on the ground plane of a microstrip transmission line and acted as a band stop filter. However, the sensitivity of double-ring and single-ring CSRRs has not been systematically investigated. 

This paper presents a high-sensitivity microwave sensor based on an interdigital-capacitor-shaped defected ground structure (IDCS-DGS) for permittivity characterization. The sensitivity of the proposed IDCS-DGS-based sensor is compared with conventional sensors based on a double-ring complementary split ring resonator (DR-CSRR), a single-ring CSRR (SR-CSRR), and a rotated single-ring CSRR (R-SR-CSRR) by measuring the shift in the resonant frequency of the transmission coefficient. All the sensors were designed and fabricated on 0.76-mm-thick RF-35 substrate and operated at 1.5 GHz under unloaded conditions. The operating frequency of 1.5 GHz was chosen because the lowest frequency above 1 GHz found in the literature for permittivity characterization using the CSSR structure ranged from 1.3 GHz to 1.7 GHz [28], and 1.5 GHz was selected for the center frequency of the lowest frequency above 1 GHz. Full-wave simulations were performed using CST Microwave Studio.

## 2. Sensor Design and Sensitivity Comparison

**Figure 1:**
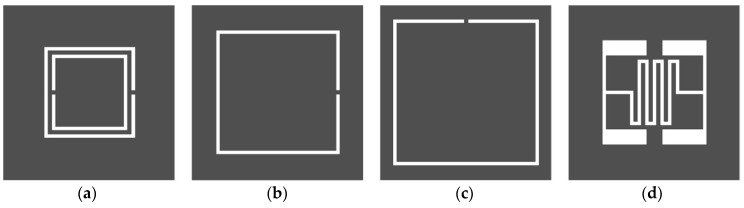
Four different resonator shapes considered for sensitivity comparison: (**a**) double-ring complementary split ring resonator (DR-CSRR); (**b**) single-ring complementary split ring resonator (SR-CSRR); (**c**) rotated single-ring complementary split ring resonator (R-SR-CSRR); (**d**) interdigital-capacitor-shaped defected ground structure (IDCS-DGS).

Figure 1 shows the four different resonator shapes that were considered for the sensitivity comparison. The dark gray regions corresponded to the conductive parts on the ground plane of a microstrip transmission line, whereas the white regions corresponded to the etched-out parts. The performance of the proposed IDCS-DGS was compared with that of a conventional DR-CSRR, SR-CSRR, and R-SR-CSRR. The four sensors were designed to resonate at 1.5 GHz to measure the dielectric property at a common frequency, because the permittivity of a material is dispersive as a function of frequency. The dimensions of the four resonators were scaled for operating at 1.5 GHz with the same slot width of 0.5 mm. The resonant frequencies of the four sensors were different because the geometric shape and location of each resonator were all different from the viewpoint of microstrip excitation. In fact, the resonant frequency of the DR-CSRR-based sensor was the lowest among the four when the outer dimensions were the same.

**Figure 2:**
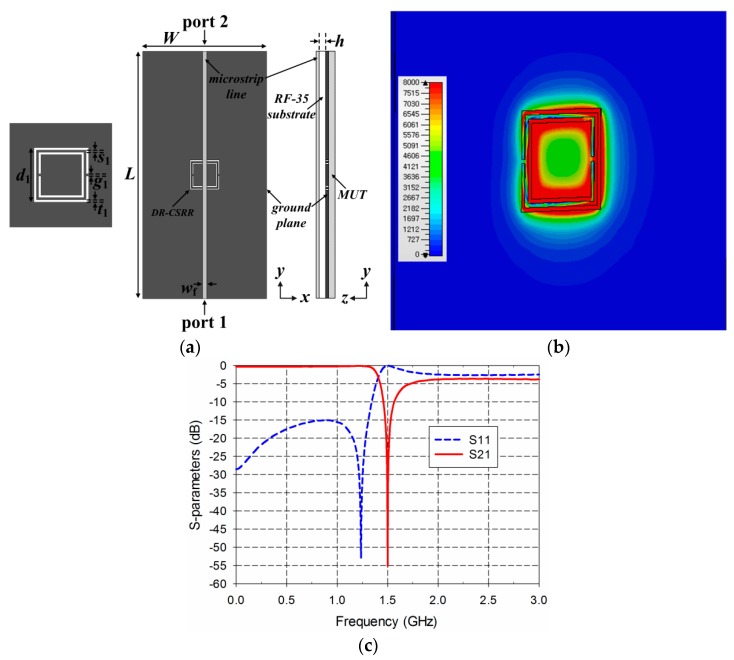
DR-CSRR-based permittivity sensor: (**a**) structure; (**b**) electric-field distribution at 1.5 GHz; (**c**) S-parameter characteristics.

Figure 2 shows the structure, electric-field distribution, and S-parameter characteristics of the DR-CSRR-based permittivity sensor. A microstrip line with a width of *w*_f_ = 1.68 mm was printed on an RF-35 substrate (*ε*_r_ = 3.5, tan *δ* = 0.0018, *h* = 0.76 mm) in order to match the 50-ohm characteristic input impedance. The width and length of the ground plane were *W* = 50 mm and *L* = 100 mm, respectively. The MUT was placed below the ground plane for loaded conditions, as shown in Figure 2a. The DR-CSRR consisted of two square split ring slots, and its dimensions were selected as *t*_1_ = 0.5 mm, *g*_1_ = 0.5 mm, *s*_1_ = 0.5 mm, and *d*_1_ = 11.92 mm to make the transmission coefficient (S_21_) resonate at 1.5 GHz under unloaded conditions. The reflection coefficient (S_11_) had a reflection zero at 1.24 GHz and a maximum value at 1.5 GHz. It can be seen that the electric fields were spread over the double-ring slots. The DR-CSRR and SR-CSRR-loaded microstrip lines were considered as a one-dimensional epsilon-negative metamaterial, and the equivalent circuit of them could be modeled as a three-element shunt LC tank [29]. The shunt LC tank circuit consisted of a series LC resonator (*L* and *C*_1_) and a capacitance element (*C*_2_) connected in parallel. In this equivalent circuit, there existed two characteristic frequencies—reflection zero frequency and transmission zero frequency. The reflection zero frequency was given by the resonance condition of the whole tank circuit, whereas the transmission zero frequency was given by the resonance condition of the series LC circuits (*L* and *C*_1_). The reflection zero frequency could be removed by adding conducting patches beside the microstrip line to fully cover the aperture of the CSRR or making the aperture structure symmetric along the center of the microstrip line. Therefore, the reflection zero frequency disappeared in the R-SR-CSRR and IDCS-DGS-based sensors because resonator geometries were symmetric along the center of the microstrip line.

**Figure 3:**
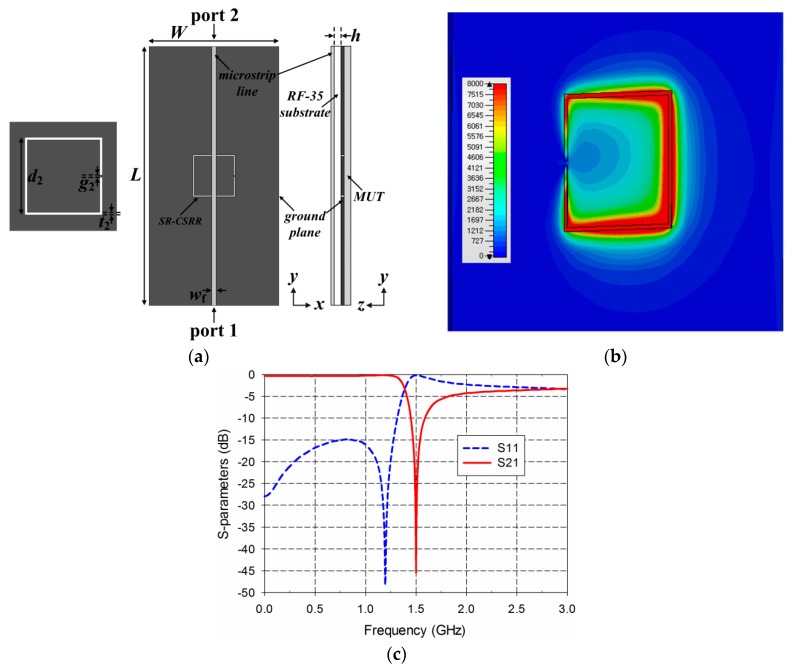
SR-CSRR-based permittivity sensor: (**a**) structure; (**b**) electric-field distribution at 1.5 GHz; (**c**) S-parameter characteristics.

Figure 3 shows the structure, electric-field distribution, and S-parameter characteristics of the SR-CSRR-based permittivity sensor. The dimensions of the microstrip line and ground plane were the same as those for the DR-CSRR. The SR-CSRR was the outer square split ring slot of the DR-CSRR, and its dimensions for its S_21_ resonance at 1.5 GHz were chosen as *t*_2_ = 0.5 mm, *g*_2_ = 0.5 mm, and *d*_2_ = 16.2 mm. In this case, the behaviors of the S-parameters were similar to those of the DR-CSRR-based sensor, and the reflection zero of S_11_ appeared at 1.19 GHz. To resonate at 1.5 GHz, the outer dimensions of the SR-CSRR became larger compared to the DR-CSRR. 

**Figure 4:**
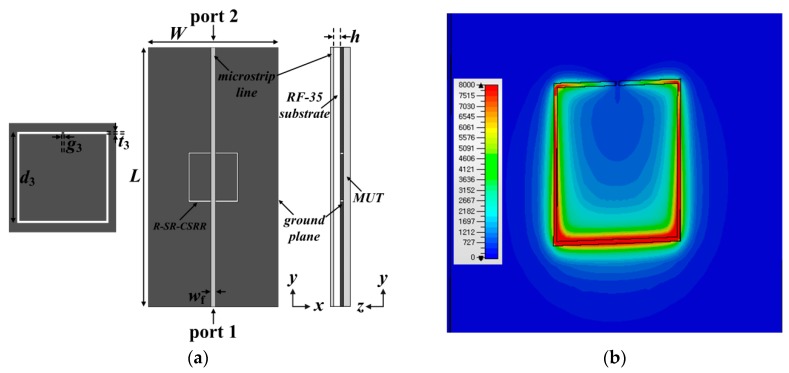

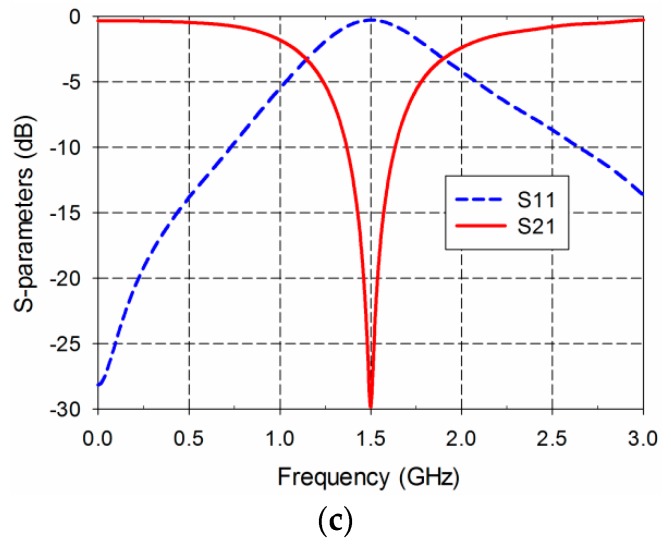
R-SR-CSRR-based permittivity sensor: (**a**) structure; (**b**) electric-field distribution at 1.5 GHz; (**c**) S-parameter characteristics.

Figure 4 presents the structure, electric-field distribution, and S-parameter characteristics of the R-SR-CSRR-based permittivity sensor. The dimensions of the microstrip line and ground plane were the same as those for the DR-CSRR. The R-SR-CSRR was rotated 90 degrees counter-clockwise compared to the SR-CSRR and looked similar to a C-shaped aperture [30], and the S_11_ reflection zero frequency disappeared. Its dimensions for S_21_ resonance at 1.5 GHz were chosen as *t*_3_= 0.5 mm, *g*_3_ = 0.5 mm, and *d*_3_ = 19.1 mm. The electric-field distributions were mainly concentrated along the non-split parts of the R-SR-CSRR. To resonate at 1.5 GHz, the outer dimensions of the R-SR-CSRR were larger compared to the DR-CSRR and SR-CSRR.

**Figure 5:**
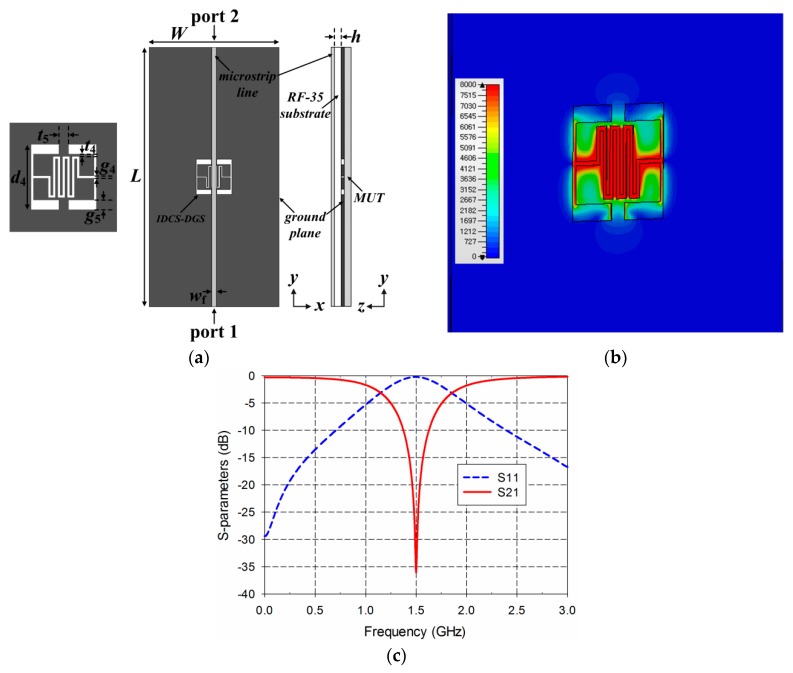
Proposed IDCS-DGS-based permittivity sensor: (**a**) structure; (**b**) electric-field distribution at 1.5 GHz; (**c**) S-parameter characteristics.

Finally, Figure 5 shows the electric-field distribution and S-parameter characteristics of the proposed IDCS-DGS-based permittivity sensor. The dimensions of the microstrip line and ground plane were the same as those for the DR-CSRR. The proposed IDCS-DGS was designed by modifying the straight ridge structure of an H-shaped aperture into an interdigital-capacitor-shaped ridge [31], and its dimensions for S_21_ resonance at 1.5 GHz were chosen as *t*_4_ = 0.5 mm, *t*_5_ = 2 mm, *g*_4_ = 0.5 mm, *g*_5_ = 2 mm, and *d*_4_ = 13.63 mm. The geometric parameters of the proposed sensor were optimized for higher sensitivity through a parametric study. Note that the outer dimensions of the IDCS-DGS were reduced compared to the SR-CSRR and R-SR-CSRR. In this case, the S_11_ reflection zero frequency disappeared, similar to the R-SR-CSRR-based sensor. The electric-field distributions were mainly concentrated on the interdigital capacitor part and this concentration area was more distributed along the microstrip line compared to the other three sensors.

**Figure 6:**
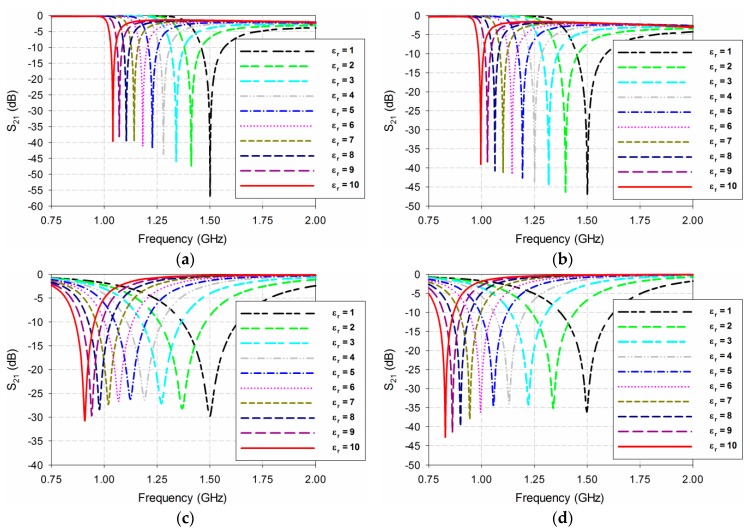
S_21_ characteristics of the four permittivity sensors for the varying permittivity of the material under test (MUT): (**a**) DR-CSRR; (**b**) SR-CSRR; (**c**) R-SR-CSRR; (**d**) proposed IDCS-DGS.

Next, the S_21_ characteristics of the four permittivity sensors were compared, as shown in Figure 6. The MUT was placed below the ground plane, and its permittivity (ε_r_) was varied from 1 to 10 with a step of 1. In this case, the loss tangent of the MUT was set as 0 like in the lossless case. The width and length of the MUT were the same as those of the ground plane, and its thickness was chosen to be 1.6 mm because the greatest thickness of the substrates available from Taconic, which was used as the MUTs, was around 1.6 mm. For the DR-CSRR-based sensor, the resonance frequency of S_21_ moved from 1.5 GHz to 1.041 GHz when ε_r_ increased from 1 to 10, whereas it shifted from 1.5 GHz to 0.997 GHz for the SR-CSRR-based sensor. The resonant frequency of S_21_ moved further from 1.5 GHz to 0.908 GHz for the R-SR-CSRR-based sensor. Finally, for the proposed IDCS-DGS-based sensor, the resonance frequency shift was maximized from 1.5 GHz to 0.829 GHz. Table 1 summarizes the resonant frequencies of the S_21_ responses with different permittivities of the MUT for the four permittivity sensors.

**Figure 7:**
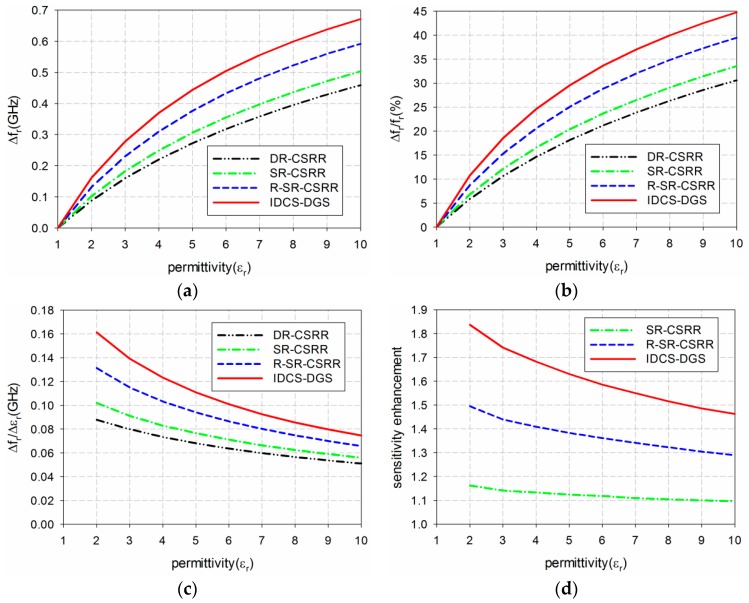
Sensitivity comparison of the four permittivity sensors: (**a**) frequency shift (Δf_r_ (GHz)); (**b**) percent relative frequency shift (Δf_r_/f_r_ (%)); (**c**) sensitivity(Δf_r_/Δε_r_ (GHz)); (**d**) sensitivity enhancement compared to the DR-CSRR-based sensor.

The sensitivity of the four permittivity sensors was analyzed from the results shown in Figure 6 and Table 1. The shift in the resonant frequency (Δf_r_ = f_r_unloaded_ − f_r_loaded_) of S_21_ is plotted in Figure 7a. Figure 7b shows the percent relative frequency shift (Δf_r_/f_r_unloaded_(%)) of the four permittivity sensors. Next, the sensitivity of the sensors was defined as the ratio of the resonant frequency change to the permittivity change (S = Δf_r_/Δε_r_ = |(f_r_unloaded_ − f_r_loaded_)|/|(ε_r_unloaded_ − ε_r_loaded_)|) [32], and is plotted in Figure 7c. Figure 7d presents the sensitivity enhancement of the SR-CSRR, R-SR-CSRR, and proposed IDCS-DGS-based sensors compared to the DR-CSRR-based sensor, which is the ratio of the sensitivity of the SR-CSRR, R-SR-CSRR, and proposed IDCS-DGS-based sensors to that of the DR-CSRR-based sensor (S__SR-CSRR,R-SR-CSRR,IDCS-DGS_/S__DR-CSRR_). When the MUT was loaded below the ground plane, it affected the total capacitance related to the resonators etched on the ground plane and the S_21_ resonant frequency was a nonlinear function of the resonator related capacitance [20]. It was observed that the sensitivity was not constant as a function of the permittivity, and it became higher for the smaller value of the permittivity. In other words, the sensitivity of the sensors decreased monotonically when the permittivity increased. 

For example, when the permittivity of the MUT was ε_r_ = 2, the resonant frequency shifts Δf_r_ of the DR-CSRR, SR-CSRR, R-SR-CSRR, and proposed IDCS-DGS-based permittivity sensors were 0.088 GHz, 0.102 GHz, 0.131 GHz, and 0.161 GHz, whereas the percent relative frequency shifts Δf_r_/f_r_unloaded_ (%) were 5.9%, 6.8%, 8.75%, and 10.8%, respectively. In this case, the sensitivity was the same as the resonant frequency shift because the difference in the permittivity (Δε_r_) was 1. The sensitivity of the SR-CSRR, R-SR-CSRR, and proposed IDCS-DGS-based sensors increased to 1.16, 1.50, and 1.84 times higher compared to the DR-CSRR-based sensor, as shown in Figure 7d.

However, when the permittivity of the MUT was increased to ε_r_ = 10, the resonant frequency shifts Δf_r_ of the DR-CSRR, SR-CSRR, R-SR-CSRR, and proposed IDCS-DGS-based permittivity sensors were 0.459 GHz, 0.503 GHz, 0.592 GHz, and 0.671 GHz, whereas the percent relative frequency shifts Δf_r_/f_r_unloaded_ (%) were 30.6%, 33.6%, 39.5%, and 44.8%, respectively. The sensitivities of the DR-CSRR, SR-CSRR, R-SR-CSRR, and proposed IDCS-DGS-based sensors were 0.051 GHz, 0.056 GHz, 0.066 GHz, and 0.075 GHz. The sensitivity enhancements of the SR-CSRR, R-SR-CSRR, and proposed IDCS-DGS-based sensors were 1.10, 1.29, and 1.46 compared to the DR-CSRR-based sensor. We can conclude that the sensitivity of the proposed IDCS-DGS-based sensor was moderately higher than that of the conventional DR- and SR-CSRR-based sensors.

## 3. Experiment Results and Discussion

**Figure 8:**
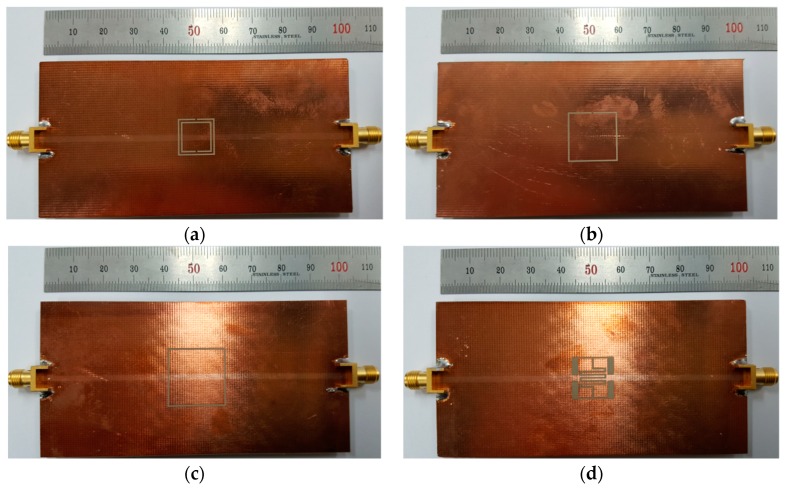
Photographs of the fabricated permittivity sensors: (**a**) DR-CSRR; (**b**) SR-CSRR; (**c**) R-SR-CSRR; (**d**) proposed IDCS-DGS.

**Figure 9:**
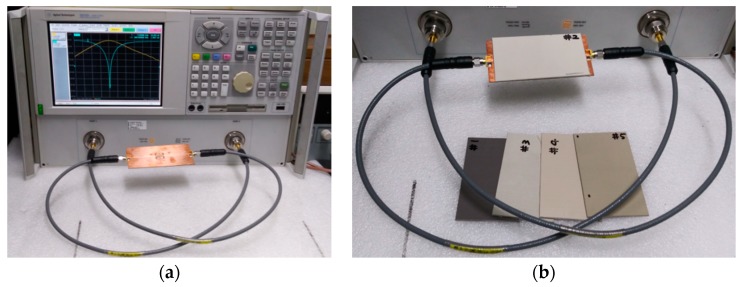
Experimental setup: (**a**) unloaded; (**b**) loaded.

Prototypes of the four permittivity sensors described in the previous section were fabricated on an RF-35 substrate (*ε*_r_ = 3.5, tan *δ* = 0.0018, *h* = 0.76 mm), as shown in Figure 8. The S-parameter characteristics of the fabricated sensors were measured using an Agilent N5230A network analyzer, and a photograph of the experimental setup is shown in Figure 9. Five different standard dielectric MUTs from Taconic were tested for the sensitivity comparison. The MUTs had permittivity values ranging from 2.17 to 10.2, and their permittivity, loss tangent, and thickness from the data sheet [33] are summarized in Table 2. 

**Figure 10:**
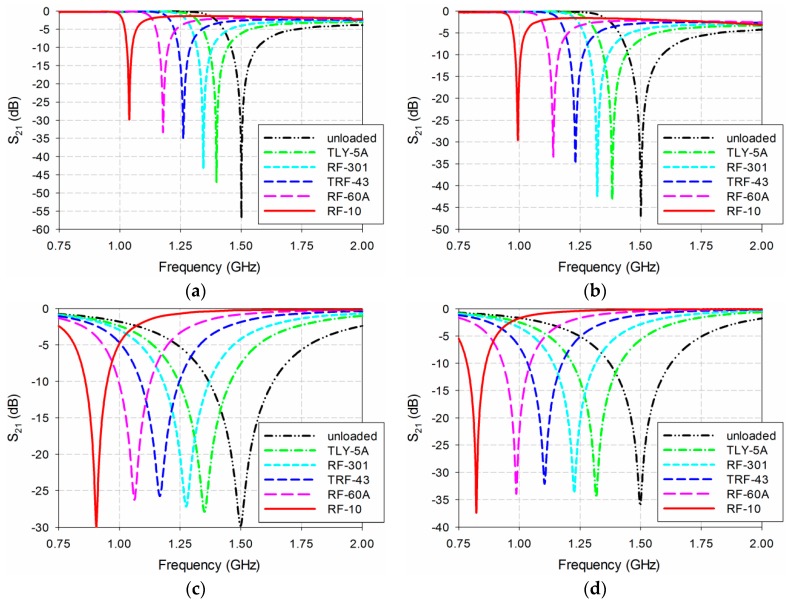
Simulated S_21_ characteristics of the four permittivity sensors for MUTs in Table 2: (**a**) DR-CSRR; (**b**) SR-CSRR; (**c**) R-SR-CSRR; (**d**) proposed IDCS-DGS.

The S_21_ characteristics of the four permittivity sensors were first simulated again with the five MUTs in Table 2 placed below the ground plane, as shown in Figure 10. The length of the MUTs was slightly reduced to 90 mm because there existed two protruding parts in the SMA connectors for soldering in the two ports of the fabricated sensors, as shown in Figure 9b. For the DR-CSRR-based sensor, the resonant frequency of S_21_ moved from 1.399 GHz for TLY-5A (ε_r_ = 2.17) to 1.039 GHz for RF-10 (ε_r_ = 10.2). In the case of the SR-CSRR-based sensor, the frequency shifted from 1.384 GHz for TLY-5A to 0.994 GHz for RF-10. For the R-SR-CSRR-based sensor, the resonant frequency of S_21_ moved from 1.351 GHz for TLY-5A to 0.905 GHz for RF-10, while it shifted from 1.317 GHz to 0.823 GHz for the proposed sensor. Table 3 shows the resonant frequencies of the simulated S_21_ responses for the four permittivity sensors.

**Figure 11:**
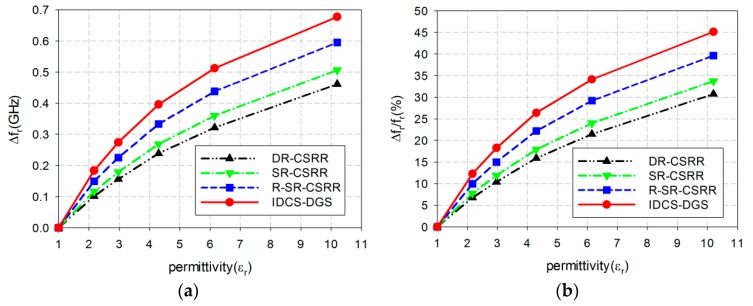

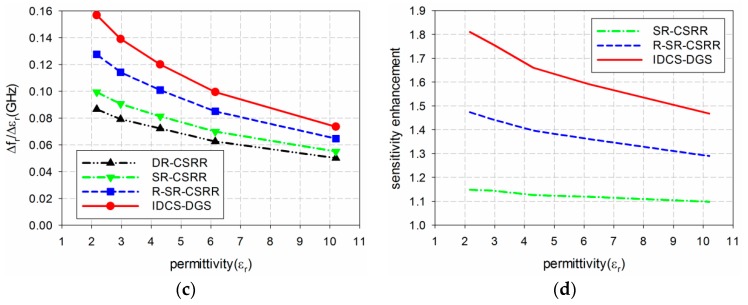
Simulated sensitivity comparison of the four permittivity sensors: (**a**) frequency shift (Δf_r_ (GHz)); (**b**) percent relative frequency shift (Δf_r_/f_r_ (%)); (**c**) sensitivity(Δf_r_/Δε_r_ (GHz)); (**d**) sensitivity enhancement compared to the DR-CSRR-based sensor.

Figure 11 compares the sensitivity of the four permittivity sensors when five MUTs are loaded. When TLY-5A (ε_r_ = 2.17) was used as the MUT, the resonant frequency shifts Δf_r_ of the DR-CSRR, SR-CSRR, R-SR-CSRR, and proposed sensors were 0.101 GHz, 0.116 GHz, 0.149 GHz, and 0.183 GHz, and the percent relative frequency shifts Δf_r_/f_r_unloaded_ (%) were 6.8%, 7.8%, 10.0%, and 12.3%, respectively. The sensitivities of the DR-CSRR, SR-CSRR, R-SR-CSRR, and proposed IDCS-DGS-based sensors were 0.087 GHz, 0.099 GHz, 0.128 GHz, and 0.157 GHz. The sensitivity enhancements of the SR-CSRR, R-SR-CSRR, and proposed IDCS-DGS-based sensors were 1.15, 1.47, and 1.81 compared to the DR-CSRR-based sensor. When RF-10 (ε_r_ = 10.2) was used as the MUT, the respective Δf_r_ values were 0.461 GHz, 0.506 GHz, 0.595 GHz, and 0.677 GHz, and the values of Δf_r_/f_r_unloaded_ (%) were 30.8%, 33.8%, 39.7%, and 45.2%. The sensitivities of the DR-CSRR, SR-CSRR, R-SR-CSRR, and proposed IDCS-DGS-based sensors were 0.050 GHz, 0.055 GHz, 0.065 GHz, and 0.074 GHz. The sensitivity enhancements of the SR-CSRR, R-SR-CSRR, and proposed IDCS-DGS-based sensors were 1.10, 1.29, and 1.47 compared to the DR-CSRR-based sensor. Therefore, the trends of Δf_r_, Δf_r_/f_r_unloaded_ (%), sensitivity, and sensitivity enhancement were similar to those of the lossless case in Figure 6, and the only difference was the loss tangent. Since the loss tangent of the MUT mainly influences the magnitude level and quality factor of the S_21_ characteristics of the sensors [22] and the loss tangent of the MUTs is relatively low—ranging from 0.0009 to 0.0035—, the effect of the loss tangent on the S_21_ resonant frequencies would be relatively insignificant. 

**Figure 12:**
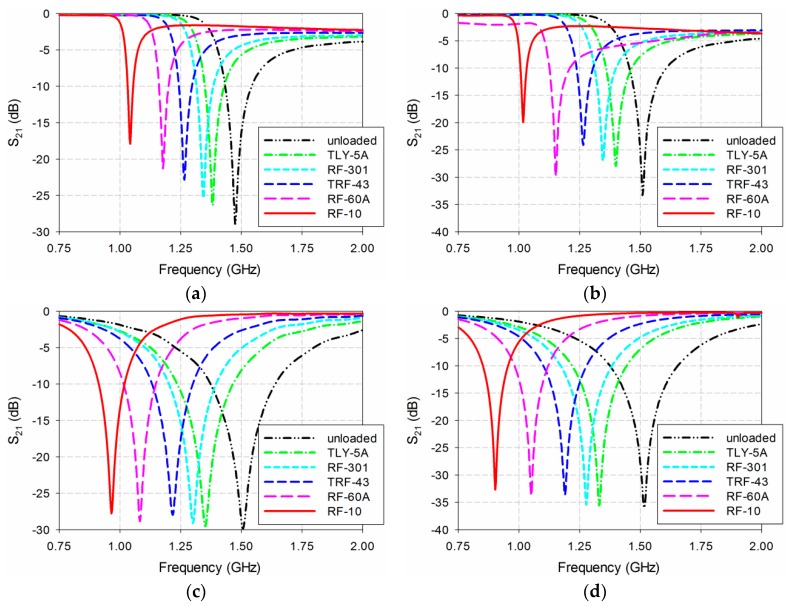
Measured S_21_ characteristics of the four permittivity sensors for MUTs in Table 2: (**a**) DR-CSRR; (**b**) SR-CSRR; (**c**) R-SR-CSRR; (**d**) proposed IDCS-DGS.

Next, the simulated sensitivity of the four sensors was validated by measuring their S_21_ characteristics when the five MUTs in Table 2 were placed below the ground plane, as shown in Figure 12. The length of the MUTs was slightly reduced to 90 mm. For the unloaded case, the S_21_ resonant frequencies of the DR-CSRR, SR-CSRR, R-SR-CSRR, and proposed IDCS-DGS sensors were 1.475 GHz, 1.510 GHz, 1.508 GHz, and 1.518 GHz, respectively, which were close to the simulated resonant frequency of 1.5 GHz. The respective errors compared to the simulated resonant frequency for the unloaded case were 1.7%, 0.7%, 0.5%, and 1.2%, which might have been caused by errors in fabrication and measurement.

For the DR-CSRR-based sensor, the resonant frequency of S_21_ moved from 1.383 GHz for TLY-5A to 1.043 GHz for RF-10, while it moved from 1.400 GHz for TLY-5A to 1.018 GHz for RF-10 for the SR-CSRR-based sensor. For the R-SR-CSRR-based sensor, the resonant frequency of S_21_ moved from 1.353 GHz for TLY-5A to 0.965 GHz for RF-10. Finally, the frequency shifted from 1.333 GHz to 0.903 GHz for the proposed IDCS-DGS-based sensor. Table 4 summarizes these results.

**Figure 13:**
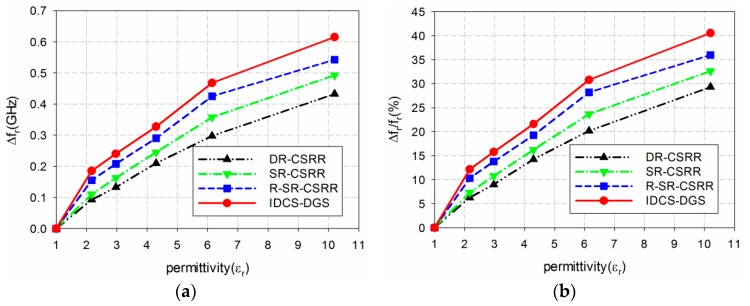

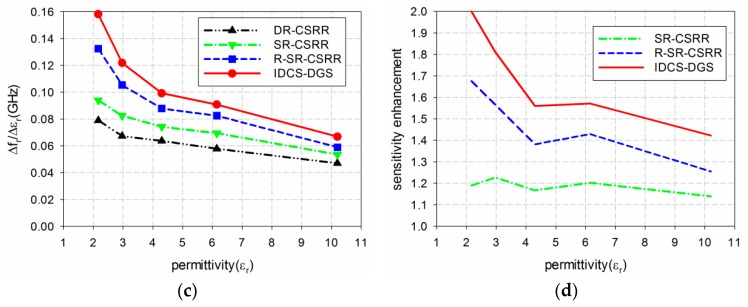
Measured sensitivity comparison of the four permittivity sensors: (**a**) frequency shift (Δf_r_ (GHz)); (**b**) percent relative frequency shift (Δf_r_/f_r_ (%)); (**c**) sensitivity (Δf_r_/Δε_r_ (GHz)); (**d**) sensitivity enhancement compared to the DR-CSRR-based sensor.

The measured sensitivity of the four permittivity sensors is compared in Figure 13a,b. The measured resonant frequency shifts Δf_r_ of the DR-CSRR, SR-CSRR, R-SR-CSRR, and proposed IDCS-DGS sensors for TLY-5A MUT were 0.093 GHz, 0.110 GHz, 0.155 GHz, and 0.185 GHz, while the measured percent relative frequency shifts Δf_r_/f_r_unloaded_ (%) were 6.2%, 7.3%, 10.3%, and 12.2%, respectively. The measured enhancement in sensitivity for TLY-5A was 5.9%. The sensitivities of the DR-CSRR, SR-CSRR, R-SR-CSRR, and proposed IDCS-DGS-based sensors were 0.079 GHz, 0.094 GHz, 0.133 GHz, and 0.158 GHz. The sensitivity enhancements of the SR-CSRR, R-SR-CSRR, and proposed IDCS-DGS-based sensors were 1.19, 1.68, and 2.00 compared to the DR-CSRR-based sensor. When RF-10 was used as the MUT, the resonant frequency shifts Δf_r_ of the DR-CSRR, SR-CSRR, R-SR-CSRR, and proposed IDCS-DGS sensors were 0.433 GHz, 0.493 GHz, 0.543 GHz, and 0.615 GHz, while the percent relative frequency shifts Δf_r_/f_r_unloaded_ (%) were 29.8%, 32.6%, 36.0%, and 40.5%, respectively. The sensitivities of the DR-CSRR, SR-CSRR, R-SR-CSRR, and proposed IDCS-DGS-based sensors were 0.047 GHz, 0.054 GHz, 0.059 GHz, and 0.067 GHz. The sensitivity enhancements of the SR-CSRR, R-SR-CSRR, and proposed IDCS-DGS-based sensors were 1.14, 1.25, and 1.42 compared to the DR-CSRR-based sensor.

The differences in the measured and simulated percent relative frequency shift Δf_r_/f_r_unloaded_ (%) for the low permittivity MUT of TLY-5A were low at 0.1% to 0.5%, but the differences increased to 1.0% to 4.7% for RF-10, which has a high permittivity. The reason for the error in the permittivity value for RF-10 increasing compared to that for TLY-5A could be measurement error such as imperfect contact or a small air gap when the MUT was placed below the ground plane of the sensors. In other words, an air gap between the ground plane and the MUT causes a relatively large error for high permittivity [22].

## 4. Conclusions

A high-sensitivity planar microwave sensor based on an IDCS-DGS has been designed, fabricated, and tested for permittivity characterization at 1.5 GHz. The sensitivity of the proposed sensor was systematically compared with that of conventional CSRR-based sensors using five MUTs from Taconic. The measured sensitivity of the proposed sensor was two times higher for TLY-5A (ε_r_ = 2.17) and 1.42 times higher for RF-10 (ε_r_ = 10.2) compared to the conventional DR-CSRR-based sensor. The proposed high-sensitivity microwave sensor is expected to be used for the permittivity characterization of planar solid and microfluidic liquid materials or for wireless sensing of biological samples. It also might be applicable as an environmental sensing unit for chipless radio frequency identification (RFID) sensors for measuring temperature, humidity, and other characteristics.

## Figures and Tables

**Table 1:** Resonant frequencies of S_21_ responses for the four permittivity sensors in GHz.

Sensor Type	ε_r_ = 1	ε_r_ = 2	ε_r_ = 3	ε_r_ = 4	ε_r_ = 5	ε_r_ = 6	ε_r_ = 7	ε_r_ = 8	ε_r_ = 9	ε_r_ = 10
DR-CSSR	1.5	1.412	1.340	1.280	1.228	1.182	1.142	1.105	1.071	1.041
SR-CSSR	1.5	1.398	1.318	1.251	1.194	1.145	1.103	1.064	1.028	0.997
R-SR-CSSR	1.5	1.367	1.270	1.190	1.124	1.067	1.019	0.977	0.941	0.908
Proposed IDCS-DGS	1.5	1.339	1.222	1.130	1.056	0.996	0.945	0.901	0.863	0.829

**Table 2:** Permittivity, loss tangent, and thickness of the five MUTs.

No.	MUT	ε_r_	tan δ	Thickness
1	TLY-5A	2.17 ± 0.02	0.0009@10 GHz	1.58 mm
2	RF-301	2.97 ± 0.07	0.0012@1.9 GHz	1.52 mm
3	TRF-43	4.3 ± 0.15	0.0035@10 GHz	1.63 mm
4	RF-60A	6.15 ± 0.15	0.0028@10 GHz	1.52 mm
5	RF-10	10.2 ± 0.3	0.0025@10 GHz	1.52 mm

**Table 3:** Simulated resonant frequencies of S_21_ responses for the four permittivity sensors in GHz.

Sensor Type	Unloaded (ε_r_ = 1)	TLY-5A (ε_r_ = 2.17)	RF-301 (ε_r_ = 2.97)	TRF-43 (ε_r_ = 4.3)	RF-60A (ε_r_ = 6.15)	RF-10 (ε_r_ = 10.2)
DR-CSSR	1.5	1.399	1.344	1.262	1.178	1.039
SR-CSSR	1.5	1.384	1.322	1.232	1.14	0.994
R-SR-CSSR	1.5	1.351	1.275	1.167	1.062	0.905
Proposed IDCS-DGS	1.5	1.317	1.226	1.104	0.988	0.823

**Table 4:** Measured resonant frequencies of S_21_ responses for the four permittivity sensors in GHz.

Sensor Type	Unloaded (ε_r_ = 1)	TLY-5A (ε_r_ = 2.17)	RF-301 (ε_r_ = 2.97)	TRF-43 (ε_r_ = 4.3)	RF-60A (ε_r_ = 6.15)	RF-10 (ε_r_ = 10.2)
DR-CSSR	1.475	1.383	1.343	1.265	1.178	1.043
SR-CSSR	1.510	1.400	1.348	1.265	1.153	1.018
R-SR-CSSR	1.508	1.353	1.300	1.218	1.083	0.965
Proposed IDCS-DGS	1.518	1.333	1.278	1.190	1.050	0.903
